# Supplementary material for: Trialling an optimised social groups intervention in services to enhance social connectedness and mental health in vulnerable young people (TOGETHER): Study protocol for a feasibility randomised controlled trial
Source: PLoS One. 2023 Aug 15;18(8):e0288676. doi: 10.1371/journal.pone.0288676 (PMC10426917; doi:10.1371/journal.pone.0288676)
Supplement: S2 File — (DOCX) [file pone.0288676.s002.docx]

### **Supporting Information File 2:**

### **Eligibility Criteria and Recruitment Processes for**

### **Intervention Providers and Practitioner Survey Respondents**

**Title:** Trialling an Optimised social Groups intervention in services to Enhance social connecTedness and mental Health in vulnERable young people (TOGETHER): Study protocol for a feasibility randomised controlled trial

Table of Contents

[**Supplementary Information File 2** 1](#_Toc138961980)

[**1.** **Intervention provider inclusion criteria** 2](#_Toc138961981)

[**2.** **Practitioner implementation survey inclusion criteria** 2](#_Toc138961982)

[**3.** **Recruitment and consent methods for intervention providers** 2](#_Toc138961983)

[**4.** **Recruitment and consent methods for implementation survey respondents** 2](#_Toc138961984)

### **Intervention provider inclusion criteria**

- Currently employed with a service-user facing practitioner role, in one of the Sussex, Kent or Surrey based services who have agreed to be involved specifically in the G4H trial component of this study, or be a member of the study research team with relevant experience
- Agree to be involved in the delivery of the G4H intervention as an intervention provider in this study to participating service-users accessing the relevant service
- Agree to receive the relevant training by the research team to deliver the G4H intervention as an intervention provider in this study
- Agree to having the relevant permission from their service manager (or relevant member of staff) to undertake the activity involved in this study as an intervention provider

### **Practitioner implementation survey inclusion criteria**

- Currently employed with a service-user facing practitioner role in an NHS or non-NHS service that involves supporting young people aged 16-25 years

1. **Recruitment and consent methods for intervention providers**

A member of the research team invites (either in-person or via work email) practitioners who are working in the organisations who have agreed to be involved in the trial aspect of the study to become intervention providers. The researcher shares the relevant participant information sheet (PIS), either as a paper, online and/or electronic copy. After a minimum of 24 hours, the researcher then invites interested practitioners to provide informed consent. The consent form and a contact details form are completed in-person or via telephone or email, on a day and time suitable for the individual. Negotiations to release practitioners to take part in this study are between practitioners and their service manager (or relevant member of staff).

1. **Recruitment and consent methods for implementation survey respondents**

A member of the research team invites (via email, in-person, team meeting discussions or public promotion) practitioners working in NHS and non-NHS services across the UK involved in supporting young people aged 16-25 to take part in the online practitioner implementation survey. The researcher shares the relevant recruitment materials (participant information sheet and study relevant poster) to the team/service/organisation. As a part of this process, team members within relevant services/organisations may be asked to disseminate to any individual who is eligible to take part via any appropriate method as listed above. The invitation to take part in the survey provides a URL link or QR code to independently access the participant information sheet, consent form, and online survey on the web-based survey tool Qualtrics. Options are provided for potential participants to take part offline and to have the study documents sent as a posted or electronic version. Participants are required to provide written informed consent to take part.
